# The perceived impact of the ANZAED Eating Disorder Credential: perspectives of individuals with eating disorder lived experience

**DOI:** 10.1186/s40337-026-01690-y

**Published:** 2026-07-31

**Authors:** Madalyn McCormack, Tracy Shand, Janet Conti, Gabriella Heruc, Siân A. McLean, Katarina Prnjak, Rebecca Barns, Phillipa Hay

**Affiliations:** 1 School of Medicine, Translational Health Research Institute, Western Sydney University, Locked Bag 1797, Penrith, 2751 Australia; 2 School of Psychology, Translational Health Research Institute, Western Sydney University, Penrith, Australia; 3 School of Medicine, Eating Disorders and Nutrition Research Group, Translational Health Research Institute, Western Sydney University, Campbelltown, Australia; 4 School of Psychology and Public Health, La Trobe University, Melbourne, Australia; 5 Graduate School of Health, University of Technology Sydney, Ultimo, Australia; 6 Mental Health Services, Campbelltown Hospital, Campbelltown, Australia

**Keywords:** Eating disorders, Credential, Lived experience, Treatment, Mixed methods, Personalised treatment, Collaborative care

## Abstract

**Background:**

In response to individual and systemic barriers hindering the timely and effective treatment of eating disorders (EDs), the Australia & New Zealand Academy for Eating Disorders (ANZAED) introduced an eating disorder credential for clinicians in June 2022. The Credential aims to enhance treatment access, quality, and outcomes. While the Credential has been well received by stakeholders, its effectiveness from the perspective of those with lived experience of an ED needs to be more fully understood. The current study aims to explore the experiences and perspectives of people living with an ED in relation to their treatment experiences provided by Credentialed Eating Disorder Clinicians (credentialed clinicians) and non-credentialed clinicians.

**Methods:**

An exploratory mixed-methods cross-sectional study was conducted involving 100 participants with personal lived experience of an ED. Participants were recruited via Australian eating disorder services and organisations, including the ANZAED and National Eating Disorders Collaboration (NEDC) membership databases, with recruitment information distributed through their email and social media platforms. Participants completed a survey with open- and closed-ended questions on their attitudes towards the Credential and experiences of treatment by a credentialed clinician or non-credentialed clinician. Descriptive statistics and Mann–Whitney U tests were used to examine differences in attitudes, treatment experiences, and helpfulness ratings between participants who had seen credentialed and non-credentialed clinicians, alongside inductive thematic analysis of open-ended survey responses.

**Results:**

Irrespective of their own clinicians’ credentialing status, participants valued the Credential; almost two thirds identified that their most helpful treatment experience was with a credentialed clinician. Three themes were generated from open-ended survey responses that explored the treatment experiences of those with lived experience of an ED: (1) *Expertise*,* Accessibility*,* and Continuity of Care*, (2) *A Collaborative Approach*, and (3) *Personalised and Effective Treatment Delivered with Understanding*,* Compassion*,* and Respect.*

**Conclusions:**

The current study adds to the ED literature by highlighting the perceived value of the ANZAED Eating Disorder Credential by individuals with lived experience. Further, it supports existing research that identifies clinician’s expertise, a collaborative approach to treatment, and the provision of individualised treatment as key factors contributing to positive treatment outcomes. However, barriers to accessibility, including the cost of treatment and availability of credentialed clinicians, remain and need to be addressed for individuals to receive the full benefits of the Credential.

**Supplementary Information:**

The online version contains supplementary material available at 10.1186/s40337-026-01690-y.

## Abstract (plain language summary)

In June 2022, the Australia & New Zealand Academy for Eating Disorders (ANZAED) introduced the ANZAED Eating Disorder Credential to the public, to improve timely access to eating disorder treatment provided by appropriately trained and experienced clinicians. The current study explores the perspectives of those with a lived experience of an eating disorder towards the Credential and their treatment experiences with credentialed and non-credentialed clinicians. Participants valued the Credential’s existence, regardless of their own clinician’s credentialing status, expressing preference to receive treatment from a credentialed clinician, placing greater trust in their advice and believing the Credential would improve health outcomes and access. Almost two thirds of participants reported that their most helpful treatment experience was with a credentialed clinician and highlighted the importance of expertise, a collaborative approach, and tailoring treatment to the individual. Barriers to accessing care, a lack of interdisciplinary collaboration, and forced or involuntary treatment were found to be key contributors to participants’ least helpful treatment experiences, pointing to a need for ongoing improvements to ensure the Credential delivers its full potential.

**Supplementary Information:**

The online version contains supplementary material available at 10.1186/s40337-026-01690-y.

## Introduction

Eating disorders (EDs) are complex mental health conditions that often lead to significant psychological distress and substantial physical health impairment, increasing the risk of long-term medical complications and premature death [1–3]. While EDs are recognised as having one of the most significant impacts on health-related quality of life among all psychiatric disorders [4, 5], help-seeking for ED symptoms remain alarmingly low. A recent systematic review [6] indicated that merely 32% of individuals with EDs sought help from qualified health professionals, with little to no improvement in help-seeking rates in over a decade [7]. Furthermore, the average delay in help-seeking remains significant, with estimates suggesting that individuals wait 2.5 to 5.5 years from the onset of symptoms to receive effective care [6, 8]. Of those who do seek treatment, only about a third receive targeted ED interventions, and overall recovery rates remain less than 50% [6, 9]. This persistent treatment gap highlights the urgent need for improved access to appropriate care and a better understanding of the barriers to help-seeking among individuals with eating disorders.

In addition to individual barriers to the timely and appropriate treatment for EDs, systemic barriers also exist. A rapid review of 87 studies related to healthcare professionals’ assessment and diagnosis of EDs found that limited screening practices, difficulty identifying ED presentations, and inadequate ED training contributed to the low rates of detection, unmet treatment needs, and associated disease burden [10]. Additionally, despite the existence of empirically supported treatments for EDs, a shortage of clinicians trained in manualised protocols impacts their dissemination and implementation, resulting in a research-practice gap [11, 12]. Some of those who have received training have expressed reservations and concerns about using manualised-based treatments, due to the generalisability of research findings, as well as perceived rigidity and incompatibility with their own theoretical orientation [11, 13]. In contrast, Griffiths et al. [14] found that both diagnosed and undiagnosed individuals expressed a preference for established, manualised treatments over novel treatments. These challenges highlight the need for additional training and support for healthcare professionals to address perceived barriers in diagnosing and treating EDs [15].

McLean et al. [16] proposed that implementing a credentialing system for mental health and dietetic practitioners would address individual and systemic barriers that prevent the timely, safe, and effective delivery of care for individuals with EDs by increasing workforce capability, ensuring consumers were better informed about clinicians who meet minimum standards, and providing clearer referral pathways for clinicians and consumers to locate a credentialed health professional via a publicly available database. Credentialing also provides a structure for implementation of the competencies and practice and training standards relevant for ED treatment (e.g., ED workforce core competencies [17] and clinical practice and training standards; Australia & New Zealand Academy for Eating Disorders (ANZAED) [18–20]). Consequently, the ANZAED Eating Disorder Credential, which formally recognises the qualifications, training, knowledge and professional development activities of healthcare professionals in Australia who deliver ED treatments, was developed and officially launched in June 2022 [21, 22]. The Credential requires health professionals to meet specified criteria, including a minimum of two years of prior clinical experience, completion of introductory training, and treatment provision training through an National Eating Disorders Collaboration (NEDC) approved course. Once credentialed, clinicians must complete a minimum of 15 h of eating disorder relevant continuing professional development (CPD) per year. This includes at least six hours of supervision relevant to eating disorders, with a minimum of three hours of individual supervision in to maintain their Credential. Clinicians are required to keep a record of their CPD activities, which may be subject to auditing [21].

Lived experience perspectives are imperative to understanding treatment engagement, perceived barriers, and experiences of care contributing to meaningful and clinically relevant research that can improve treatment outcomes [23, 24]. While there is an increasing presence of lived experience advocacy in the ED field, only a handful of studies exist that examine the perceived helpfulness of ED treatment by those with a lived experience [25–29]. This gap may reflect challenges associated with conducting research with vulnerable populations, including ethical concerns related to potential harm or relapse risk. However, emerging evidence suggests that involvement of individuals who have a lived experience can be achieved in an ethical and valuable way [25]. Therefore, evaluating the Credential from a lived experience perspective provides an important opportunity to explore its perceived effectiveness, drawing directly on the voices of individuals living with an ED who have received treatment from credentialed and non-credentialed clinicians.

The main aim of this study was to explore the attitudes and experiences of ED treatment by ANZAED Credentialed Eating Disorder Clinicians and non-credentialed clinicians. We also aimed to explore features associated with perceived helpfulness of treatment using the Treatment Experiences Sliding Scale Rater (TESSR) [30] and test the hypothesis that treatment experiences with a credentialed clinician would be rated more favourably than treatment experiences with a non-credentialed clinician, for both experiences that were reported as most helpful overall and experiences reported as least helpful.

## Methods

### Design

The present study used an exploratory cross-sectional mixed-methods design. Analyses compared self-reported treatment experiences with credentialed clinicians and non-credentialed clinicians.

### Procedure

The Western Sydney University Human Ethics Committee approved the study (approval number: H15252).

Participants were recruited via Australian eating disorder services and organisations (Butterfly Foundation, Eating Disorders Queensland (EDQ), Eating Disorders Victoria (EDV), Inside Out Institute), and through ANZAED and NEDC membership databases. These organisations sent the recruitment information out via their mailing lists and social media platforms. The survey comprised a range of closed and open-ended self-report questions answered anonymously and disseminated via an online questionnaire hosted by Qualtrics from April – August 2023. Participants who consented and completed the survey received a reimbursement of $20 to thank them for their involvement in the study. At the end of the survey, participants were invited to express interest in participating in an interview. This paper reports only the survey findings, while a more detailed analysis of the interview data is presented in Martin et al. [31].

### Participants

A total of 100 participants, 81 who had seen a credentialed clinician and 19 who had seen a non-credentialed clinician, were included in the study. Participants were eligible to participate if they reported that they had a personal lived experience of an ED and had received eating disorder treatment by a credentialed clinician or a non-credentialed clinician either currently or in the past. The participant characteristics are shown in Table 1. Data for characteristics with small sample sizes were not reported for confidentiality reasons. Participants had a mean age in their early thirties and most identified as women. More than half of participants reported an Oceanic ethnic background, resided in a metropolitan area, were single, and had completed a university or college diploma as their highest level of education. Participants reported varied employment with the most commonly reported being full time employment, part-time/causal employment, studying and/or a combination of employment. All participants had received an eating disorder diagnosis, with anorexia nervosa being the most commonly reported diagnosis. The vast majority of all participants met or exceeded the cut-off score of 15 for clinically significant eating disorder symptoms, in the Eating Disorder Examination Questionnaire-Short (EDE-QS) [32, 33] in both participants who had seen a credentialed clinician (94.4%) and those who had seen a non-credentialed clinician (93.7%). Most participants, across both groups, also exceeded the cut off score for the anxiety subscale of the Hospital Anxiety and Depression Scale (HADS) [34], indicating clinically significant anxiety. There was a clinically significant difference observed in the depression subscale of the HADS, whereby participants who had seen a non-credentialed clinician were less likely to experience elevated levels of depression than participants who had seen a credentialed clinician (Z = -2.335, *p* = .020).

**Table 1:** Participants Characteristics

Feature	Treating health professional	
	Credentialed clinician (*n* = 81)	Non-credentialed clinician (*n* = 19)
	Mean ± SD	
**Age**	31.1 (9.2)	32.1 (10.9)
** n (%)**		
**Gender**		
Women	69 (85.2%)	15 (78.9%)
Men or Other	12 (14.7%)	*
**Cultural background** ^1^		
Oceanic	50 (61.7%)	11 (57.9%)
European	13 (16%)	*
Other (e.g. Asian, Oceanic & European, North American)	18 (22.2%)	*
**Relationship status**		
Married/Living as married	22 (27.2%)	*
Single	54 (66.7%)	11 (57.9%)
**Geographical location**		
Metropolitan	65 (80.2%)	13 (68.4%)
Regional & Rural	16 (19.7%)	*
**Highest level of education**		
University or college diploma	57 (70.4%)	12 (63.2%)
Year 12 High School Certificate	14 (17.3%)	*
Other (e.g. Year 10 High School, Trade/Apprenticeship)	10 (12.3%)	*
**Employment status**		
Employed full-time	24 (29.6%)	*
Employed part-time/casually	14 (17.3%)	*
Currently studying	12 (14.8%)	*
Other (e.g. studying and working part time)	31 (38.3%)	*
**ED diagnosis** ^1^		
Currently experiencing symptoms	65 (80.2%)	*
Previously experienced symptoms		10 (52.6%)
Anorexia Nervosa	64 (79%)	14 (73.7%)
Bulimia Nervosa	19 (23.5%)	*
Binge Eating Disorder	14 (17.3%)	*
Other Specified Feeding or Eating Disorder	17 (21%)	*
Other Diagnosis^2^	12 (14.8%)	*
**Symptom Scores**		
EDE-QS Score ≥ 15^3^	67 (94.4%)	14 (93.7%)
HADS Score^3^		
Anxiety ≥ 8	63 (90%)	12 (80.1%)
Depression ≥ 8	40 (57.1%)	5 (33.4%)

^*^﻿*n* < 10 were not included in the table to protect participant confidentiality, therefore some cells have been left blank

^1^These are not exclusive categories

^2^‘Other’ included Rumination, Pica, Avoidant/Restrictive Food Intake Disorder, Unspecified Feeding or Eating Disorder and Atypical Anorexia

^3^These survey items were not set up to force a response and some participants chose not to respond

### Measures

The survey (see Additional File 1) consisted of questions that asked all participants about their:

- * Demographics*. Participants were asked to indicate their age, gender, level of education, cultural and ethnic background, residential location (including the Australian state or territory they reside in and the geographical setting of their primary residential location), marital status and employment.
-  *ED history*. Participants were asked about their ED history including the year their ED symptoms began, the status of their symptoms (currently experiencing, previously experienced, never experienced), and ED diagnosis.
- *Treatment history*. All participants were asked about their treatment history including the type and duration of treatment received, professionals and/or specialists they received treatment from, geographic setting of care, and wait time to access treatment.
- *Experience using ‘Find a treatment provider’ searchable directory.* Participants were asked to detail their experiences (e.g. access, clinician preferences, general user experience) using the ‘Find a treatment provider’ searchable directory of the credentialing system.
- *Treatment experience with a Credentialed Eating Disorder Clinician.* Participants were asked if they had ever received eating disorder treatment from an ANZAED Credentialed Eating Disorder Clinician. Those who indicated they had seen a credentialed clinician either currently or in the past were asked about their treatment history with a credentialed clinician including the type of treatment and professionals and/or specialists seen, geographic setting of care, wait time to access treatment, and length of treatment.
-  *Attitudes toward the ANZAED Eating Disorder Credential.* All participants rated six items regarding their attitudes towards the Credential on a 6-point Likert scale from 0 (strongly disagree) to 5 (strongly agree). An example item is “I would prefer receiving treatment from a credentialed clinician.” Responses to items were analysed separately.
- * Perceived barriers to seeking and accessing treatment.* All participants were asked about the perceived barriers that affected their access or use of outpatient or inpatient ED treatment using a 5-point scale ranging from 1 (*no impact at all*) to 5 (*extremely impactful*). Participants were also provided with the option to provide a free text response if there were other barriers not listed.
- *Most positive and least positive treatment experience* Participants were asked to identify their most and least helpful treatment experience, and for each experience to indicate if it was with a Credentialed Eating Disorder Clinician (response options were yes, no, not sure), how old they were at the time, and characteristics of the treatment. Then, separately for the most and least helpful treatment experiences, participants were asked to keep those experiences in mind and respond to a series of 11 items using the Treatment Experiences Sliding Scale Rater (TESSR) adapted from the Eating Disorder Treatment Experiences Survey (EDTES) developed by authors JC and PH [28, 30] and inspired by the Session Rating Scale [35]. Each item was anchored by two opposing statements, such as whether the treatment approach “Did NOT work for me/ Worked well for me; Did NOT assist me in recovering from the eating disorder/Assisted me in recovering from the eating disorder”. Participants indicated their perceptions of their experience by rating each item on a sliding scale from − 50 (negative anchor) to 50 (positive anchor). Scores for the most helpful and least helpful experiences were calculated as the median of item scores (range − 50 to 50). Higher scores indicated more positive experiences. Internal consistency in this sample was excellent (McDonald’s omega = 0.89) for the most helpful treatment experience and 0.96 for the least helpful treatment experience. Participants were also invited to respond to a series of open-ended questions about their most positive and least positive treatment experiences. For their most positive treatment experience, participants were asked to reflect on the most helpful aspects, the least helpful aspects, and what they would have liked more of. For their least positive treatment experience, participants were asked to describe the least helpful aspects, any aspects they found helpful, and whether there was anything they would have liked more of in that treatment.

Participants completed validated measures of eating disorder and depression/anxiety symptoms. The former were assessed with the EDE-QS [32]. This scale has 12 items that measure eating disorder symptoms over the past 7 days using a 4-point response scale (ranging from 0 [*0 days*] to 3 [*6–7 days*]). A total score is calculated from the sum of item responses (range 0–36). Higher scores indicate more severe ED symptoms. Scores greater than 15 indicate increased risk of an eating disorder being present [33]. Internal consistency for this sample was excellent (McDonald’s omega= 0.89). Depression and anxiety symptoms were assessed with the HADS [34], a 14 item self-report scale assessing symptoms in the past week. Participants respond to items on a 4-point scale (ranging from 0 to 3) and responses are summed to form a scale total. Scores of at least 8 indicate clinical severity. Internal consistency in this sample was good for depression (McDonald’s omega = 0.84) and anxiety (McDonald’s omega = 0.86).

### Data analysis

Data processing and statistical analyses were performed using IBM SPSS Statistics Version 29.0.2.0. Data were cleaned and inspected for normality of distributions. The skewness and kurtosis results and an inspection of histograms found that scores on sliding scale questions were non-normally distributed, requiring non-parametric tests for analyses [36]. Descriptive statistics using frequencies and proportions were used to explore treatment experiences and perceptions of treatment by a credentialed or non-credentialed clinician. Mann Whitney U-tests were used to examine if between groups differences in perceptions of treatment and differences in ratings of helpfulness of treatment were statistically significant.

Inductive thematic analysis [37, 38] was selected to explore treatment experiences through open-ended survey responses. In light of limited existing literature on the evaluation of the Credential from a lived experience perspective, this approach allowed for the identification of implicit themes from the participant’s explicit language without imposing pre-existing frameworks, with the assumption that language constructs a version of lived experience and meaning-making processes [39]. Written survey responses were collated into a Microsoft Excel Spreadsheet and were read and re-read by MM and TS to gain familiarity. Responses were then coded, and initial themes were generated from the codes. The initial themes were further developed, refined, and defined. Emergent themes were discussed between authors MM, TS, JC and PH to refine overarching themes (see Additional File 2 for reflexivity statement). Exemplar quotes (see Additional File 3) from a broad range of participants who completed the open-ended survey questions were included to illustrate themes and subthemes.

## Results

### Quantitative analyses

#### Attitudes towards the credential

When asked about their attitudes towards the Credential, participants who had seen a non-credentialed clinician were less likely to agree that seeing a credentialed clinician would impact the success of therapy (Z = -3.122, *p* = .002).   Interestingly, all participants expressed a preference for receiving treatment from a credentialed clinician, regardless of whether they had seen a credentialed clinician or a non-credentialed clinician. Both groups also indicated that they placed greater trust in the advice of a credentialed clinician, valued the existence of the Credential, and agreed that the Credential will make it easier for people to access specialised care and improve the health outcomes of eating disorder patients. However, examination of the distribution of responses from the two groups showed that where participants agreed with favourable perceptions of the Credential, those who had received treatment from a credentialed clinician tended to strongly endorse these favourable perceptions relative to participants who had received treatment from a non-credentialed clinician. For example, more than double the proportion of participants who received treatment from a credentialed clinician (44.3%) than participants who received treatment from a non-credentialed clinician (20%) strongly agreed that they would prefer to receive treatment from a credentialed clinician. Table 2 presents participants responses to questions about their attitudes towards the Credential.

**Table 2:** Participant responses to questions about their attitudes towards the Credential

Survey questions and response options	Response distribution (%) of participants who have seen a credentialed clinician (*n* = 70)	Response distribution (%) of participants who have seen a non- credentialed clinician (*n* = 15)	Mann- Whitney U Z (*p*)
In terms of the success of therapy, it makes no difference whether I receive treatment from a credentialed or non-credentialed clinician.			− 3.12 (0.002)
* Strongly disagree*	17.1	0	
* Disagree*	24.3	6.7	
* Somewhat disagree*	21.4	13.3	
* Somewhat agree*	17.1	33.3	
* Agree*	11.4	20.0	
* Strongly agree*	8.6	26.7	
I would prefer receiving treatment from a credentialed clinician over a non-credentialed clinician			− 1.36 (0.173)
* Strongly disagree*	4.3	6.7	
* Disagree*	4.3	6.7	
* Somewhat disagree*	2.9	0	
* Somewhat agree*	15.7	20.0	
* Agree*	28.6	46.7	
* Strongly agree*	44.3	20.0	
I place greater trust in the advice of a Credentialed Eating Disorder Clinician than a non-credentialed clinician			− 0.73 (0.466)
* Strongly disagree*	4.3	0	
* Disagree*	4.3	13.3	
* Somewhat disagree*	5.7	13.3	
* Somewhat agree*	14.3	6.7	
* Agree*	28.6	33.3	
* Strongly agree*	42.9	33.3	
I value the existence of a Credential that recognises the expertise and training of clinicians and requires a commitment to engagement in continual professional development and supervision specific to the treatment of eating disorders.			− 0.73 (0.466)
* Strongly disagree*	1.4	6.7	
* Disagree*	0	0	
* Somewhat disagree*	0	0	
* Somewhat agree*	12.9	13.3	
* Agree*	30.0	33.3	
* Strongly agree*	55.7	46.7	
The Credential will make it easier for people with an eating disorder to readily access specialised care.			− 1.56 (0.118)
* Strongly disagree*	2.9	0	
* Disagree*	2.9	6.7	
*Somewhat disagree*	2.9	6.7	
* Somewhat agree*	22.9	26.7	
* Agree*	20.0	40.0	
* Strongly agree*	48.6	20.0	
The Credential will improve the health outcomes of eating disorder patients.			− 0.88 (0.381)
*Strongly disagree*	1.4	0	
* Disagree*	0	0	
* Somewhat disagree*	5.7	6.7	
* Somewhat agree*	21.4	26.7	
* Agree*	28.6	40.0	
* Strongly agree*	42.9	26.7	

### Treatment history and experiences

Of the 81 participants who had seen a credentialed clinician, 67.9% reported receiving treatment currently and 32.1% reported receiving treatment in the past. Most commonly they reported finding a credentialed clinician through a General Practitioner (GP) or other health professional (55.1%), referral from family, friends, or a carer (14.1%), the ’Find a treatment provider’ searchable directory on the Connected website (7.7%) or through independent advertising, social media, and other/unknown (23.1%). From the time they first began to seek treatment from a credentialed clinician, they reported a median wait time for treatment of 2 months (IQR = 1-2.75) and experienced a median treatment duration of 13 months (IQR = 5–36), including both participants who were still receiving treatment at the time of the survey and those who had completed treatment.

Treatment usually occurred in a metropolitan setting (85.9%) and was accessed in person (91%) and/or via Telehealth (67.9%). The most common credentialed professionals or specialists that people with a lived experience received treatment from were dietitians (75.6%), psychologists (59%), and psychiatrists (32.1%).

Most participants who received ED treatment from a non-credentialed clinician (*n* = 19) reported receiving treatment in the past (68.4%), while the remaining 31.6% of participants reported that they were currently receiving treatment. The most commonly seen clinicians were dietitians (73.7%), psychologists (78.9%), psychiatrists (57.9%) and general practitioners (84.2%). The median wait time to access treatment was 2 months (IQR = 1–3) and most treatment occurred in a metropolitan setting (73.7%).

Participants were invited to describe their most (*n* = 89) and least (*n* = 87) helpful treatment experiences. More than half (60.7%) of participants specified that their most helpful treatment experience was with a credentialed clinician, whilst almost one third of participants noted that it was with a non-credentialed clinician (27%). These proportions should be interpreted descriptively rather than comparatively as we did not capture whether participants had seen a credentialed clinician only, a non-credentialed clinician only, or both.

The most commonly reported helpful treatments experienced with a credentialed clinician were cognitive behavioural therapy (CBT; 27.8%), other psychological interventions (e.g. schema therapy, Acceptance and Commitment Therapy (ACT), Psychotherapy; 37%), and dietetic treatments (20.4%). Whereas, for non-credentialed clinicians three quarters of participants reported that their most helpful treatment experienced was other psychological interventions (e.g. individual therapy, schema therapy, narrative therapy; 75%).

When asked about their least helpful treatment experience, participants were more likely to report that this treatment was with a non-credentialed clinician (46%) than a credentialed clinician (27.6%). Those participants who indicated that their least helpful treatment experience was with a credentialed clinician most commonly described experiencing inpatient and/or residential treatments (33.3%), dietetic treatment (20.8%) or other treatments (20.8%).

Similarly, participants who indicated that their least helpful treatment experience was with a non-credentialed clinician also commonly reported experiencing inpatient/residential treatment (30%) and other psychological interventions (e.g. Family Based Therapy, Eye Movement Desensitization and Reprocessing (EMDR), group therapy;35%). Table 3 outlines treatment-related characteristics for participants who rated credentialed and non -credentialed clinician treatment experiences including their age at the time of treatment, whether treatment was entered voluntarily, and mood at the time of treatment.

**Table 3:** Clinical features of participants with an eating disorder who rated credentialed and non -credentialed clinician treatment experiences

Feature	Most helpful (positive) treatment experience				Least helpful (positive) treatment experience		
	CC (*n* = 54)	NCC (*N* = 24)		CC (*n* = 24)		NCC (*N* = 40)	
	Mean ± SD			Mean ± SD			
**Age (years)**	27.7 (8.7)	29.2 (8.8)		26.0 (9.5)		25.3 (7.2)	
	**n (%)**		**Man** **n-Whitney U ** ***Z (p)***	**n (%)**			**Mann- Whitney U** ***Z (p)***
**Entered treatment voluntarily**			− 0.459 (0.646)				− 0.80 (0.425)
Yes	51 (94.4%)	22 (91.7%)		19 (79.2%)		28 (70%)	
**Mood at the time of treatment**			− 0.562 (0.574)				**− 2.42 (0.016)**
Extremely negative	8 (14.8%)	4 (16.7%)		5 (20.8%)		21 (52.5%)	
Negative	17 (31.5%)	5 (20.8%)		11 (45.8%)		11 (27.5%)	
Neither negative or positive	10 (18.5%)	6 (25%)		1 (4.2%)		4 (10%)	
Positive	19 (35.2%)	7 (29.2%)		7 (29.2%)		4 (10%)	
Extremely positive		2 (8.3%)					

Using a Mann-Whitney U test, the median helpfulness scores were compared between the most helpful and least helpful treatment experiences of those participants who had seen credentialed and non-credentialed clinicians (see Table 4). There were no significant differences in participants’ most helpful treatment experiences between those participants who saw a credentialed clinician and those who saw a non-credentialed clinician. However, ratings of the participant’s least helpful treatment experience were significantly different at the 0.01 level, whereby the overall helpfulness of the treatment, the treatment approach working well and assisting in recovery, meeting their hopes and expectations, shifting their relationship with difficult emotions, and the therapist addressing their concerns were consistently rated lower by participants whose least helpful treatment experience was with a non-credentialed clinician relative to participants whose least helpful treatment experience was with a credentialed clinician.

**Table 4:** Most and least helpful treatment experiences with a credentialed and non-credentialed clinician

Treatment Experiences Ratings (TESSR)	Most helpful (positive) treatment experience with a:			Least helpful (positive) treatment experience with a:		Mann-Whitney U
	Credentialed Clinician (*n* = 54)	Non-Credentialed Clinician (*N* = 24)	Mann-Whitney U	Credentialed Clinician (*n* = 24)	Non-Credentialed Clinician (*N* = 40)	
	Median (IQR)		Z (*p*)	Median (IQR)		Z (*p*)
When I sought this treatment, I thought change was important or possible	6.5 (− 35.5–29.3)	22.0 (− 35.8–40.3)	− 1.42 (0.156)	3.0 (− 30.3–19.5)	− 24.0 (− 49.5–17.8)	− 1.38 (0.168)
The treatment approach worked well for me	30.0 (18.8–36.3)	26.0 (15.5–39.8)	− 0.37 (0.709)	− 20.0 (− 45.8–0.8)	− 48.0 (−50.0 to − 26.3)	**− 2.73 (0.006)**
The treatment approach met my hopes and expectations	27.5 (15.0–39.3)	28.0 (15.5–43.0)	− 0.20 (0.845)	− 16.5 (− 39.8–13.3)	− 48.5 (-50.0 to − 21.3)	**− 3.34 (< 0.001)**
The treatment approach assisted me in shifting my relationship with difficult emotions	29.0 (13.8–37.5)	34.5 (22.8–40.8)	− 1.21 (0.227)	− 11.0 (− 33.3–11.0)	− 45.0 (− 50.0 – 19.0)	**− 2.73 (0.006)**
The treatment approach assisted me in recovering from the eating disorder	26.5 (15.0–35.5)	25.5 (10.5–40.0)	− 0.14 (0.892)	− 20.0 (− 37.0–4.3)	− 41.0 (− 50.0 to − 12.3)	**− 2.57 (0.010)**
The treatment approach took into consideration my treatment preferences	32.5 (13.3–45.3)	35.0 (19.3–49.5)	-0.67 (0.504)	-19.5 (-47.8 − 10.0)	− 40.0 (− 50.0 – − 14.3)	− 2.21 (0.027)
The treatment approach gave me freedom to make my own choices around change	27.0 (12.5–46.3)	35.5 (25.0–50.0)	− 1.14 (0.254)	− 14.0 (− 41.8–27.0)	− 27.5 (− 50.0 to − 9.0)	− 2.15 (0.031)
I felt understood by the therapist	39.0 (28.0–50.0)	42.0 (28.3–49.8)	− 0.06 (0.956)	-7.5 (-32.8–8.8)	− 40.5 (− 50.0 – 7.0)	− 2.47 (0.013)
The therapist addressed my concerns	37.0 (21.0–50.0)	34.0 (22.8–44.0)	− 0.86 (0.391)	− 19.0 (-29.0–7.8)	− 39.5 (− 50.0 to −10.0)	**− 2.71 (0.007)**
The therapist did instil hope for recovery	46.0 (27.0–50.0)	44.5 (29.3–50.0)	− 0.21 (0.831)	− 12.0 (-21.5–18.8)	− 29.0 (− 49.8–8.5)	− 1.98 (0.047)
Overall, how helpful did you think your eating disorder treatment was?	34.0 (21.0–42.3)	36.0 (22.5–43.0)	− 0.25 (0.803)	− 19.0 (-37.3–15.3)	− 42.0 (-50.0 to − 25.3)	**− 3.28 (0.001)**

Median scores reflect participants’ perception of the helpfulness of the experience with responses ranging from − 50 (negative anchor, e.g., the treatment approach did NOT work for me) to  50 (positive anchor, e.g., the treatment approach worked well for me)

### Qualitative analysis

Results from the qualitative analysis complemented quantitative findings by using a parallel convergent design where both strands are collected concurrently and analysed separately before merging results [40]. This allowed for a deeper understanding of the comparison of the aspects of treatment by credentialed and non-credentialed clinicians that participants with personal lived experience found helpful and unhelpful and what they would like more of in their treatment. The thematic analysis identified three main themes: (1) Accessing consistent, expert care (2) Working together in treatment and (3) Feeling seen and supported in care.

### Theme 1: Accessing consistent, expert care

Participants with personal lived experience of an ED identified the importance of their clinician having ED specific expertise, treatment being accessible, and highlighted the value of continuity of care.

### Theme 1.1: Clinician expertise

When recalling their most helpful treatment experience, ED specific expertise, evidence-based treatment, and lived experience were identified as key aspects that contributed to the helpfulness of the treatment approach, irrespective of whether they saw a credentialed or a non-credentialed clinician. Some participants identified evidence-based therapies that were utilized in the most helpful treatment experience (e.g. enhanced cognitive behavioural therapy). While others expressed the need for clinicians to tailor treatment to their own individual needs considering their capacity, comorbidities, and readiness to change (discussed further in Theme 3).

"My psychologist specialises in eating disorders and is so knowledgeable. She used the ED specific CBT [CBT-E] and is now exploring other types of therapy as I also have ASD [Autism Spectrum Disorder]."

Many participants valued working with clinicians who had specific ED expertise, including the ability to look beyond appearance and trauma informed practice: *“she understood that I was not ok despite not being underweight”.*

Conversely, participants who received treatment from a non-credentialed clinician were more likely to identify a lack of ED expertise as a key feature of their least helpful treatment experience. Participants described feeling invalidated, judged, and criticized and reported that perceived clinician inexperience led to delays in accessing appropriate treatment:

"The first psychiatrist I was referred to did not take any of my concerns seriously and said I did not fit the criteria for an eating disorder as I was a regular weight. She told me I probably just need a holiday to deal with stress and I would come back feeling better."

"Under qualified psychologist who was critical, often would not talk about my eating disorder, did not push me to challenge my eating disorder, criticised my parents constantly and created an unsafe space for my family, did not take concerns seriously, felt belittled and misunderstood."

Clinicians with lived experience were described as supporting participants to feel heard and understood, and they also instilled hope that recovery was possible. Some participants spoke specifically about working in an ongoing capacity with a clinician or peer mentor:

"My therapist struggled with an eating disorder and had fully recovered. She understood and could relate to everything I was saying and still found a way out. She challenged me endlessly and listened to me without judgement. It was the first time I had hope."

Whilst one participant spoke about the impact of “*having recovered individuals come and talk to inpatients about their stories”* noting that this is something they would like more of in their treatment.

### Theme 1.2: Barriers in accessing ongoing and consistent care

In relation to continuity of care, barriers to treatment were identified as one of the least helpful aspects of treatment by participants, regardless of whether they had seen a credentialed or non-credentialed clinician. The cost of treatment, strict admission criteria, and long wait times, particularly during the COVID-19 pandemic, were problematic for participants and the frequency of appointments were often limited to what was financially feasible or due to treatment system constraints rather than what was required for effective treatment. Participants spoke often about feeling as though they could benefit from more intensive support and/or more frequent appointments, highlighting the need for additional support between appointments. Some participants who had seen a credentialed clinician also noted their desire for “*more focus on follow up support post therapy”* to support them as they transitioned to less intense treatment (e.g. after discharge from an inpatient admission). While another participant who saw a non-credentialed clinician described the need for more public awareness of what services are available.

### Theme 2: Working together in treatment

Participants with lived experience discussed the need for a collaborative approach to treatment to feel empowered to have choice over their treatment goals, actively participate in their recovery, and have their voices heard. When reflecting on their most helpful treatment experience, both participants who saw a credentialed clinician and those who saw a non-credentialed clinician highlighted the importance of autonomy to have “*input around the treatment*”, *“choice over topics discussed”*, their voice “*heard and respected”*, and the ability to make changes at a pace they were comfortable with (e.g. *“make changes slowly”*). A few participants who saw a credentialed clinician for their most helpful treatment experience also noted that collaboration between client and clinician is one that requires a *“sense of partnership”*, a strong therapeutic rapport and involves a delicate balance of empowering the client to make their own decisions, when appropriate, and the clinician holding firm boundaries and taking control when safety is at risk or the “*eating disorder is loud*”.

"Whilst I had decision making opportunities, my clinician at times made different choices which I may not have agreed with but were in the best interest for my survival, overall health, and long-term recovery. My clinician was honest about risks and dangers of engaging in ED behaviours and was honest with me about treatment I was being provided and worked with me even when I was not 100% agreeable with the approach. I still felt heard and respected."

This extract highlights the imperative for clinicians to balance openness about the real effects of an ED whilst also prioritising respect and the voice of the person living with an ED. Participants who saw a credentialed clinician also noted the need for clinicians to be flexible in their sessions to ensure that there is enough time set aside to discuss what the client’s bring to the session rather than focusing on their own set agenda. Forced and/or involuntary treatment was emphasized by participants when describing the least helpful aspect of their treatment experience, regardless of their clinicians’ credentialing status, often resulting in participants feeling like they had lost their power and control, they were not heard or respected, and their treatment goals and/or approach did not align with their needs: *“Involuntary treatment was extremely difficult without a sense of autonomy*,* choice*,* power*,* control*,* etc.”*

When reflecting on their most helpful treatment experience, participants who had seen a credentialed clinician highlighted the importance of collaboration between their multidisciplinary team. Whereas a few participants who had seen a non-credentialed clinician identified this as a key aspect missing in their helpful treatment experience and something they would have liked more of (e.g. *“For my psychologist to properly liaise with my GP”).*

### Theme 3: Feeling seen and supported in care

Participants reported that treatment tailored to their individual circumstances and delivered with understanding, compassion, and respect significantly shaped the quality of their treatment experience.

### Theme 3.1: Treatment tailored to the individual

When reflecting on their most helpful treatment experience, participants valued both credentialed and non-credentialed clinicians who took the time to understand their specific needs and provided *“individualised care”* rather than relying on a one-size-fits-all approach: “*People who cared and listened and didn’t just follow a textbook or manual.”* It was also important to be seen as a “whole person” whose underlying issues and comorbidities were considered and for clinicians to meet them where they were at in terms of their motivation and readiness to change.

In contrast, participants who described their least positive experiences with both credentialed and non-credentialed clinicians described being provided with generic interventions that did not consider their personal circumstances. One participant shared: *“No real attempt to understand my unique circumstances and was a variation of a just-eat approach without nuanced attention to motivation*,* capacity*,* and disability issues impacting my ability to do this.”* Others described feeling dismissed, alienated, judged, punished, disrespected, or misunderstood in their struggles:

"I felt that my digestive illnesses or irritations were dismissed."

"I needed understanding. I felt so alone, and he made me feel like an outcast to a community I had expected to understand me or maybe empathise."

"I felt like I was being punished every second of every day, in addition to living in total fear of gaining a single gram of weight."

"It strips the patient of their individuality and autonomy. My deep psychological needs were never addressed as the only thing spoken about was food which only fueled the anorexia."

Although participants described this as their least helpful treatment experience, many still identified beneficial aspects when asked if there was anything helpful about this treatment. They noted improvements in physical health, increased medical stability and safety, relief from symptoms, greater insight, and skill development. These benefits were reported across both credentialed and non-credentialed clinicians.

### Theme 3.2: Compassionate, understanding and respectful care

The therapeutic relationship was perceived by those participants who saw a credentialed clinician as an imperative part of effective treatment. Many participants described working with credentialed clinicians who were understanding, compassionate, respectful, non-judgemental and caring and noted that the therapeutic relationship instilled hope and created a safe therapeutic environment to support recovery:

“*the manner in which the clinician approached working with me - compassion and understanding without demands or threats”.*

“The non-judgement and the caring nature of all involved has been paramount in me not giving up what little hope I have of recovery.”

*“nothing could be effective without a feeling of safety with the therapist”* *.*

These extracts exemplify the responses of many of the participants who described working with credentialed clinicians. Features of the therapeutic relationship included understanding, compassionate, respectful, non-judgmental and caring, instilling hope, and a safe therapeutic environment to support recovery.

## Discussion

The recent introduction of the ANZAED Eating Disorder Credential for ED clinicians necessitates the need for an evaluation of its effectiveness to justify its continued use. McLean et al. [16] proposed that a credentialing system for ED clinicians must address the individual and systemic barriers that hinder the timely, safe, and effective delivery of care. In line with this, the main aim of this study was to explore the perspectives of individuals with a lived experience of an ED, regarding their attitudes and treatment experiences with non-credentialed and credentialed clinicians.

The study found that participants who saw a credentialed clinician were more likely to agree that seeing a credentialed clinician would impact on the success of therapy compared to those who had not seen credentialed clinician. However, irrespective of their own clinician’s credentialing status, participants valued the existence of the Credential. They reported a preference for receiving treatment from a credentialed clinician and indicated greater trust in the advice provided by credentialed clinicians compared to non-credentialed clinicians. Participants also endorsed the view that the Credential improves health outcomes for individuals living with an ED and facilitates access to specialised care. These attitudes support the proposition that the Credential may enhance the safety and effectiveness of the delivery of care for individuals with EDs [16]. However, free-text responses describing participants’ actual treatment experiences offered a more nuanced perspective on this assumption, as described below.

In terms of treatment experiences, participants were more likely to report that their most helpful treatment had been delivered by a credentialed clinician, whereas their least helpful treatment was more often delivered by a non-credentialed clinician. Although, there were no significant differences in treatment experience ratings of their most helpful treatment between participants who saw credentialed versus non-credentialed clinicians, significant differences emerged in ratings of the least helpful treatment. Specifically, participants who saw a non-credentialed clinician rated their least helpful treatment significantly lower on measures of overall helpfulness, effectiveness of the treatment approach, extent to which the treatment met their hopes and expectations, perceived improvement in their relationship with difficult emotions, and the extent to which the therapist addressed their concerns. These findings suggest that differences between credentialed and non-credentialed clinicians may be more pronounced in negative treatment experiences than in positive ones. It is important to note that participants who reported their least helpful treatment was delivered by a non-credentialed clinician also reported significantly lower mood at the time of treatment, which may have contributed to these differences in treatment experience. Nevertheless, it is possible the credentialling of clinicians may result in less negative experiences through improved skills and also improved health literacy, reduced stigma and ‘countertransference’ responses from health care providers [41]. Reducing the occurrence of negative treatment experiences may also aid treatment engagement and reduce the incidence of longstanding illness which has been found to be associated with greater number of episodes of treatment but poorer subjective ratings of their treatment experiences [30].

When reflecting on their most helpful treatment experiences, participants emphasised the importance of clinicians, both credentialed and non-credentialed, using evidence-based interventions and having expertise in eating disorders as well as comorbid conditions that may maintain the disorder (e.g., anxiety, depression, trauma). They also highlighted their preference for care that was tailored to their own individual circumstances and delivered with genuine understanding, compassion, and respect. For some participants, this experience was enhanced by clinicians with lived experience, who fostered a sense of being deeply understood and instilled hope that recovery was possible. This highlights that recovery and treatment effectiveness extends beyond symptom reduction and medical stabilization, encompassing broader experiences such as the development of confidence, a sense of autonomy, and the instillation of hope. These findings synergise with the recognition that personalised care will be increasingly expected and is crucial to achieving better outcomes in eating disorders [42].

A collaborative approach to treatment, in which participants experienced autonomy, choice, and a sense of control over their recovery, was identified as another key component of helpful treatment experiences with credentialed clinicians. Forced and involuntary treatment where participants lacked control and choice were key themes in their least helpful treatment experienced. Whereas, supporting autonomy in ED treatment has been associated with increased self-motivation and engagement, leading to improved treatment outcomes [43]. Participants spoke about wanting to be met at their current level of motivation and readiness to change, taking steps towards recovery that felt achievable and manageable and having the flexibility to adapt treatment if needed. However, they acknowledged that when safety was at risk, a strong therapeutic rapport enabled clinicians to hold firm boundaries and assume greater responsibility, whilst also ensuring they felt held and supported.

Consistent with Bray et al. [44], participants valued interdisciplinary collaboration where at a minimum their treatment team included a dietitian, mental health professional, and a GP. They emphasised the importance of communication within the treatment team and fostering autonomy within appropriate limits. These findings align with our survey data, in which the most commonly seen credentialed and non-credentialed clinicians were dietitians, psychologists, and medical practitioners (including psychiatrists and GPs). Participants emphasised the value of collaboration amongst their treatment team and noted that a lack of interdisciplinary collaboration was a key contributor to negative treatment experiences. This supports Heafala et al. [45] who explored the lived experience perspectives of consumers and carers of dietetic care for EDs, and found that personalisation, delivered with empathy and collaboration, were desired factors in treatment delivery.

Although the Credential was introduced to improve access to safe and effective ED care, participants who had seen both credentialed and non-credentialed clinicians reported that barriers to treatment, including cost, access, and availability, played a substantial role in shaping their least helpful treatment experiences. Some participants who had seen non-credentialed clinicians highlighted that a clinician’s lack of expertise contributed to delays in accessing appropriate treatment in a timely manner and that there was a lack of awareness of the services available. It is possible that the study being conducted in the early stages of credentialing implementation, where the number of credentialed clinicians is low, may explain this outcome. Alternatively, with many participants retrospectively recalling their treatment experiences, delays in treatment access may also reflect extended wait-times due to the high demand for eating disorder treatment that persisted post COVID-19 [46].

This study has several limitations. First, the sample size for participants who reported receiving care from a non-credentialed clinician was small (*n* = 19) and convenience sampling with predominantly well-educated female participants with a diagnosis of anorexia nervosa who self-selected to participate limits heterogeneity and the generalisability of data to all who have received care from credentialed and non-credentialed clinicians. The cross-sectional design also restricts the interpretation of data and exploration of a causal link between clinician credentialing and the helpfulness of treatment. However, cross-sectional study designs are indicated when conducting exploratory research where patterns of relationships are unknown, which is the case in this study of the recently launched ED credential [47]. Participants were also relied upon to recall their treatment experiences accurately; therefore, data may be subject to recall bias. Importantly, most participants (67.9%) who reported seeing a credentialed clinician were still receiving treatment at the time of the survey and therefore may have experienced less recall bias compared to the majority of participants (68.4%) whose treatment with a non-credentialed clinician had occurred in the past. Additionally, because our survey did not capture whether participants had received treatment exclusively from credentialed clinicians, non-credentialed clinicians, or both, the overall proportions reported for whether participants’ most helpful and least helpful treatment experiences were with a credentialed or non-credentialed clinician should be interpreted cautiously. Some participants may not have had experiences with both types of clinician limiting direct comparison. Various unknown confounding variables, such as clinician competence and treatment modality mediating and moderating the perception of treatment experiences, may also have influenced study outcomes.

It is also acknowledged that this research was undertaken during the early stages of the Credential’s implementation and therefore does not examine treatment outcomes for participants seen by credentialed compared to non-credentialed clinicians. Future research will evaluate clinical outcomes from the credentialling with a funded project to link clinical outcome registry data with health care and other data [48]. Despite these limitations, the use of a mixed methods design was advantageous due to the ease of collection of concise responses, with the option for participants to expand on their answers through open-ended questions and a rigorous analysis of qualitative data was achieved by having several authors review the data and identify and reach a consensus on the identified themes.

## Conclusions

This study is the first to perform a mixed-methods analysis of attitudes and treatment experiences by credentialed and non-credentialed clinicians from the perspective of individuals with lived ED experience. The current study adds to the ED literature by providing evidence for the benefit of clinician credentialing in treating EDs. Regardless of whether their own clinician was credentialed, the vast majority of participants valued the Credential, expressed a preference for receiving treatment from credentialed clinicians, indicated greater trust in their advice, and endorsed the view that the Credential improves health outcomes and facilitates access to specialised care.

More than half of the helpful treatment experiences reported were delivered by credentialed clinicians who had ED specific expertise, utilised a collaborative approach, fostered autonomy, and ensured treatment approaches were evidence based and tailored to the individual. The findings also suggest that differences between credentialed and non-credentialed clinicians may be more apparent in negative treatment experiences where participants identified barriers to treatment including cost, access, and availability, a lack of interdisciplinary collaboration, and forced or involuntary treatment.

Future replication of the study using a prospective longitudinal design is important to better establish the causality of determinants in treatment helpfulness. The recruitment of males and those with an ED diagnosis other than anorexia would also improve generalisability.

## Supplementary Information

Below is the link to the electronic supplementary material.

Supplementary Material 1: Lived Experience Survey. This file contains the full survey instrument used to explore the perspectives of those with a lived experience of an eating disorder towards the Credential and their treatment experiences with credentialed and non-credentialed clinicians.

Supplementary Material 2: Author Reflexivity Statement. This file contains an author reflexivity statement detailing the clinical, academic, and methodological backgrounds of the research team and exploring how these positions influenced the data analysis and interpretation.

Supplementary Material 3: Exemplar Data Extracts for Themes Identified from Participants Qualitative Survey Responses. This file contains exemplar data extracts for themes identified from participants qualitative survey responses.

## Data Availability

The additional files contain the following data and materials for this study: Lived experience survey (Additional File 1); author reflexivity statements (Additional File 2), and exemplar data extracts for themes identified from participants qualitative survey responses (Additional File 3). In line with the ethics approvals for this study, the datasets used and/or analysed during the current study are not publicly available as they contain information that could compromise the privacy and confidentiality of research participants.

## Abbreviations

ACT: Acceptance and Commitment Therapy
ANZAED: Australian & New Zealand Academy for Eating Disorders
CBT: Cognitive Behavioural Therapy
CBT-E: Enhanced Cognitive Behavioural Therapy
CPD: Continuing Professional Development
ED: Eating Disorder
EDE-QS: Eating Disorder Examination Questionnaire – Short
EDTES: Eating Disorder Treatment Experiences Survey
EDQ: Eating Disorders Queensland
EDV: Eating Disorders Victoria
EMDR: Eye Movement Desensitization and Reprocessing
GP: General Practitioner
HADS: Hospital Anxiety and Depression Scale
NEDC: National Eating Disorder Collaboration
TESSR: Treatment Experiences Sliding Scale Rater
