# Supplementary material for: The perceived impact of the ANZAED Eating Disorder Credential: perspectives of individuals with eating disorder lived experience
Source: J Eat Disord. 2026 Jul 31;13(Suppl 1):299. doi: 10.1186/s40337-026-01690-y (PMC13428425; doi:10.1186/s40337-026-01690-y)
Supplement: Supplementary file 1 — Supplementary Material 1: Lived Experience Survey. This file contains the full survey instrument used to explore the perspectives of those with a lived experience of an eating disorder towards the Credential and their treatment experiences with credentialed and non-credentialed clinicians. [file 40337_2026_1690_MOESM1_ESM.pdf]

ADDITIONAL FILE 1: Lived Experience Survey

1. Demographics

Please answer the following general questions about you.

1.1. Which option best describes your gender?

- Man
- Woman
- Prefer not to say
- Other (Please specify)
    - Free text response

1.2. What is your age in years?

- Free text response

1.3. What is your cultural and ethnic background?

- Oceanian (e.g., Australian, New Zealand, Polynesian)
- European (e.g., British, Irish, Western European, Northern European)
- Asian (e.g., Chinese, Japanese)
- Middle Eastern (e.g., Arab, Egyptian)
- North American (e.g., United States of America, Canadian)
- Central and South American (e.g., Hispanic)
- Sub-Saharan (e.g., Central and West African, Southern and East African)
- Prefer not to say

1.4. In which Australian state or territory do you currently reside?

- Australian Capital Territory
- New South Wales
- Northern Territory
- Queensland

- South Australia
- Tasmania
- Victoria
- Western Australia
- None

1.5.Which geographical setting best describes your primary residential location?

- Metropolitan
- Regional
- Rural
- Remote

1.6.What is your marital status?

- Single
- Married/living as married
- Separated, divorced or widowed

1.7.What is your current employment status? Please select all that apply.

- Currently studying
- Employed full-time
- Employed part-time/casually
- Full time home duties/caring for children
- Presently not employed, seeking employment
- Presently not employed, NOT seeking employment
- Recovering from illness or injury
- Other (Please specify)
    - Free text response

1.8.What is the highest level of education you have completed to date?

- Prior to Year 10
- Year 10 high school
- Year 12 high school
- Trade certificate apprenticeship
- University or Tertiary degree or College diploma

2. Eating Disorder History

2.1. Have you ever experienced symptoms of an eating disorder? (For example, purging, restricting food intake)

- I am currently experiencing eating disorder symptoms
- I have previously experienced eating disorder symptoms
- I have never experienced eating disorder symptoms

2.2. In what year (approximately) did you first start experiencing symptoms of an eating disorder (e.g. 2016)? Please specify:

- Free text response

2.3. Have you ever been diagnosed with an eating disorder? Please select all that apply.

- No, I have not
- Anorexia Nervosa
- Bulimia Nervosa
- Binge Eating Disorder
- Other Specified Feeding or Eating Disorder (OSFED)
- Pica
- Rumination Disorder
- Avoidant/Restrictive Food Intake Disorder (ARFID)
- Unspecified Feeding or Eating Disorder

- Other (Please specify)
    - Free text response

Question only shown to participants who answered "Yes":

2.4. In what year (approximately) were you first diagnosed with an eating disorder (e.g. 2016)? Please specify.

- Free text response

3. Treatment History

3.1. Have you ever received treatment from a professional or specialist for an eating disorder?

- Yes, I am currently receiving treatment for an eating disorder
- Yes, I received treatment in the past but not currently
- No, I have never undergone treatment for an eating disorder

Questions 3.2-3.13. only shown to participants who answered "Yes":

3.2. Please indicate which professionals or specialists you received treatment from. Please select all that apply.

- Dietitian
- General Practitioner
- Occupational Therapist
- Psychiatrist
- Psychologist
- Psychotherapist
- Social Worker
- Other (Please specify)

- Free text response

3.3. Which geographical setting best describes the location you received most of their eating disorder care?

- Metropolitan
- Regional
- Rural
- Remote

3.4. From the time you began to seek treatment, how long did you have to wait to access it? (To the nearest month).

- Free text/months

3.5. Please select the type of treatment(s) you have undertaken for the eating disorder. You can select more than one option.

Please RANK the treatments in order of DURATION with 1 indicating the longest treatment then 2, 3, 4 etc. If you have only experienced one treatment for an eating disorder, just mark 1 in the box.

- Individual Therapy
- Family Based Therapy
- Inpatient Therapy
- Group Therapy
- Nutritional Counselling/Physical Health CounsellingMedical/Psychiatric Treatment
- Other (Please specify)
    - Free text response
- I do not know what type of therapy I received

3.6. Please select which of the following "Individual Psychological Therapies" you have experienced (if known). Please select all that apply.

- Cognitive Behavioural Therapy (CBT)
- Motivational Interviewing (MI)\
- Behaviour Therapy
- Dialectical Behaviour Therapy (DBT)
- Narrative Therapy
- Mindfulness Based Therapy
- Acceptance and Commitment Therapy (ACT)
- Psychotherapy
- Psychodynamic Therapy
- Other (Please specify)
    - Free text response
- I do not know what type of therapy I received

3.7. Please select which of the following "Family Therapies" you have experienced. Please select all that apply.

- Maudsley Family Based Treatment (FBT)
- Other Family Therapy (Please specify)
    - Free text response

3.8. Please select which of the following "Inpatient Therapies" you have experienced. Please select all that apply.

- Eating Disorders Inpatient Treatment
- General medical ward
- Other (Please specify)

    - Free text response

3.9.Please select which of the following "Group Therapies" you have experienced. Please select all that apply to you.

- Dialectical Behaviour Therapy
- Skills Focused Groups
- Education Focused Group
- Eating Disorders Group

3.10. Please select which of the following "Nutritional Counselling Services" you have experienced. Please select all that apply.

- By an accredited Practising Dietitian
- Nutritionist
- Naturopath
- Other counselling

3.11. Please select which of the following "Physical Health Counselling Services" you have experienced. Please select all that apply.

- Personal fitness trainer
- Other physical health counselling (Please specify):
    - Free text response

3.12. Please select which of the following "Medical/Psychiatric treatments" you have experienced. Please select all that apply.

- Medications (please specify type of medications)
    - Free text response
- Other (please specify)
    - Free text response

3.13 Please share with us any other treatment interventions for eating disorders that you have tried?

- Free text response

4. Experience using *Find a Treatment Provider Search* Directory

4.1. Have you ever accessed the *Find a Treatment Provider Search* Directory which contains a registry of all Credentialed Eating Disorder Clinicians: https://connected.anzaed.org.au/treatmentproviders/?

- Yes
- No
- I'm not sure

Questions 4.3-4.8 only shown to participants who answered "Yes":

4.2. Do you have a strong preference for seeing a particular type of clinician?

- Yes
- No

Question 4.2.1 only shown to participants who answered "No":

4.2.1 Can you please elaborate on the reasons why you don't have a strong preference?

4.2.2 Is it important for you to find a clinician based on any of the following? Please select all that apply.

- Demographic characteristics (e.g. age, sex, ethnicity)
- Psychological characteristics (e.g. respect for your beliefs and cultural values)
- Physical characteristics (clinician weight and body shape)

4.3. Did the search filters assist in this process? Specifically in relation to the following aspects of the treatment provider:

4.3.1. Location

- Yes
- No

4.3.2. Gender

- Yes
- No

4.3.3. Areas of interest (e.g., child and adolescence health, LGBTIQ health, Aboriginal and Torres Strait Islander health)

- Yes
- No

4.3.4. Language spoken

- Yes
- No

4.4. Are there any other filters you would like us to consider developing to make your search more customisable and tailored to your needs?

- Free text response

4.5. Are there any other filters you would like us to consider developing to make your search more customisable and tailored to your needs? =

- Free text response

4.6 Did the search directory always display potential service providers in response to your search criteria?

- Yes
- No
- I'm not sure

4.7. Did you use the clinician profile function?

- Yes
- No
- I'm not sure

Question 4.7.1 only shown to participants who answered "Yes": 4.7.1 Was the clinician profile function helpful?

  - Free text response

4.8. Did the Find a Treatment Provider Directory reduce the amount of time it took you to find a clinician?

- Yes
- No
- I'm not sure

4.9. Do you feel that the Find a Treatment Provider Directory reduced the waiting time to access treatment?

- Yes
- No
- I'm not sure

5. ANZAED Credential Clinician Treatment History

5.1. To your knowledge have you ever received eating disorder treatment from an ANZAED credentialed clinician? (If in doubt please see direct link to the Find a Treatment Provider Directory which contains a registry of all Credentialed practitioners: https://connected.anzaed.org.au/treatmentproviders/)

- Yes, I am currently receiving treatment from an ANZAED credentialed clinician

- Yes, I received treatment in the past from an ANZAED credentialed clinician but not currently
- No
- I'm not sure

5.1a. In what year did you commence treatment with a credentialed clinician? E.g. 2022

    - Free text response

Questions 5.2-5.8 only shown to participants who answered "Yes":

5.2. Please indicate which professionals or specialists you received treatment from. Please select all that apply.

- Counsellor
- Dietitian
- General Practitioner
- Mental Health Nurse
- Nurse Practitioner
- Occupational Therapist
- Psychiatrist
- Psychologist
- Psychotherapist
- Social Worker

Please answer the following questions with reference to the Credentialled Practitioner you saw for the longest period of time.

5.3. From the time you began to seek treatment from a credentialed clinician, how long (approximately) did you have to wait to access it? (to the nearest month)

- Free text response/months

5.4. How long (approximately) have you received treatment from an ANZAED credentialed clinician? (to the nearest month)

- Free text response/months

5.5. Are you still receiving this treatment?

- Yes
- No

5.6. How did you find a Credentialed Eating Disorder Clinician?

- ConnectED website
- Referral by GP or other health professional
- Referral by family, friends, carers or other people who currently or have previously been diagnosed with an eating disorder
- Independent advertising (websites, clinic brochures/posters, etc.)
- Social Media (Facebook, Instagram, Twitter)
- Webinar/Seminar
- Podcast
- Other (Please specify)
    - Free text response

5.7. Which geographical setting(s) best describes the location you received care from a credentialed clinician?

- Metropolitan
- Regional
- Rural

- Remote

5.8. Which of the following treatment format(s) did you use to receive care from a credentialed eating disorder clinician? Please select all that apply.

- In-person
- Telehealth
- Telephone
- Outpatient facility
- Inpatient facility
- Other (Please specify)
    - Free text response

6. MOST POSITIVE TREATMENT EXPERIENCE

We will now ask you some questions about the eating disorder treatment you feel was most helpful to you. Please try to answer all questions in a way that best reflects your memory of this treatment experience.

6.1. The treatment that I experienced that was most helpful is/was:

____

____

____

6.2. To the best of your knowledge please specify if this was from a Credentialed Eating Disorder Clinician? (If in doubt please see direct link to the Find a Treatment Provider

Directory which contains a registry of all Credentialed practitioners: https://connected.anzaed.org.au/treatmentproviders/)

- Yes
- No
- Not sure

6.3. Did you enter this eating disorder treatment voluntarily?

- Yes
- No

6.4. Approximately how old were you when you experienced this helpful eating disorder treatment?

- Free text response

6.5. Now, we will ask you to think back to when you had your **MOST POSITIVE** treatment experience. We will be asking some questions about how you felt, and what you were thinking at that time.

After you have read each question please drag the slider to the point that most accurately reflects your answer.

6.5.1. When I sought this treatment

	I did NOT think change was important or possible	I thought change was important or possible

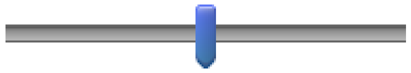

6.5.2. The treatment approach

	Did NOT work for me	Worked well for me
	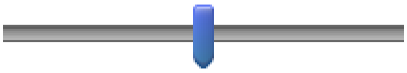	

6.5.3. The treatment approach

	Did NOT meet my hopes and expectations	Met my hopes and expectations
	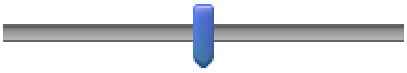	

6.5.4. The treatment approach

	Did NOT assist me in shifting my relationship with difficult emotions	Assisted me in shifting my relationship with difficult emotions
	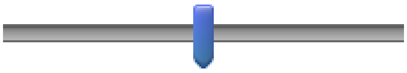	

6.5.5. The treatment approach

Did NOT assist me in recovering from the eating disorder	Assisted me in recovering from the eating disorder

6.5.6. The treatment approach

Did NOT take into consideration my treatment preferences	Took into consideration my treatment preferences

6.5.7. The treatment approach

Did NOT give me freedom to make my own choices around change	Gave me freedom to make my own choices around change

6.5.8. The treatment approach

I did NOT feel understood by the therapist	I felt understood by the therapist

6.5.9. The treatment approach

The therapist did NOT address my concerns	The therapist addressed my concerns

6.5.10. The treatment approach

The therapist DID NOT instil hope for recovery	The therapist DID instil hope for recovery

6.5.11. Overall, how helpful do you think your eating disorder treatment was?

Not helpful at all Extremely helpful

6.6. Please share with us what was the most helpful aspect about this treatment, and why?

____

____

____

6.7. Please share with us what was least helpful aspect about this treatment, and why?

____

____

____

6.8. Please share with us, based on your experience what would you have liked more of in that treatment?

____

____

____

6.9. To the best of your recollection, what was your mood at the time of this treatment?

- Extremely negative
- Negative
- Neither positive nor negative
- Positive
- Extremely Positive

7. LEAST POSITIVE TREATMENT EXPERIENCE

If you have received treatment for an eating disorder more than once, we will now ask you some questions about the eating disorder treatment you felt was **LEAST** helpful to you. Please try to answer all questions in a way that best reflects your memory of that experience.

7.1. The treatment that I experienced that was most helpful is/was:

____

____

____

7.2. To the best of your knowledge please specify if this was from a Credentialed Eating Disorder Clinician? (If in doubt please see direct link to the Find a Treatment Provider Directory which contains a registry of all Credentialed practitioners: https://connected.anzaed.org.au/treatmentproviders/)

- Yes
- No
- Not sure

7.3. Did you enter this eating disorder treatment voluntarily?

- Yes
- No

7.4. Approximately how old were you when you experienced this least helpful eating disorder treatment?

- Free text response

7.5. Now, we will ask you to think back to when you had your <u>LEAST POSITIVE</u> treatment experience. We will be asking some questions about how you felt, and what you were thinking at that time.

After you have read each question please drag the slider to the point that most accurately reflects your answer.

7.5.1. When I sought this treatment

	I did NOT think change was important or possible	I thought change was important or possible

	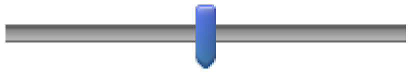

7.5.2. The treatment approach

	Did NOT work for me	Worked well for me

7.5.3. The treatment approach

Did NOT meet my hopes and expectations	Met my hopes and expectations

7.5.4. The treatment approach

Did NOT assist me in shifting my relationship with difficult emotions	Assisted me in shifting my relationship with difficult emotions

7.5.5. The treatment approach

Did NOT assist me in recovering from the eating disorder	Assisted me in recovering from the eating disorder

7.5.6. The treatment approach

Did NOT take into consideration my treatment preferences	Took into consideration my treatment preferences

7.5.7. The treatment approach

Did NOT give me freedom to make my own choices around change	Gave me freedom to make my own choices around change

7.5.8. The treatment approach

I did NOT feel understood by the therapist	I felt understood by the therapist

	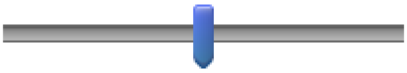

7.5.9. The treatment approach

The therapist did NOT address my concerns	The therapist addressed my concerns

	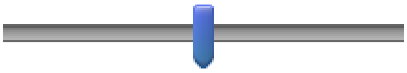

7.5.10. The treatment approach

The therapist DID NOT instil hope for recovery	The therapist DID instil hope for recovery

	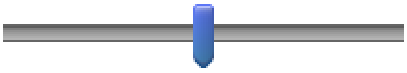

7.5.11. Overall, how helpful do you think your eating disorder treatment was?

Not helpful at all Extremely helpful

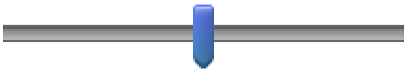

7.6. Please share with us what was the least helpful aspect about this treatment, and why?

____

____

____

7.7. Please share with us if there was anything helpful about this treatment? If so, please explain what was helpful?

____

____

____

7.8. Please share with us, based on your experience what would you have liked more of in that treatment?

____

____

____

7.9. To the best of your recollection, what was your mood at the time of this treatment?

- Extremely negative
- Negative
- Neither positive nor negative
- Positive
- Extremely Positive

8. EATING DISORDER EXAMINATION QUESTIONNAIRE - SHORT (EDE-QS) (Derived from Fairburn & Beglin, 2008)

We are interested to hear about whether you currently experience eating disorder symptoms. The following questions are concerned with past **WEEK/7 days)** only. Please read each question carefully. Please answer all of the questions. Please only choose one answer for each question. Thank you.

Remember that the questions only refer to the past **WEEK/7 days)** only.

On how many of the past **7days**….

8.1. Have you been deliberately trying to limit the amount of food you eat to influence your weight or shape (whether or not you have succeeded)?

- 0 days
- 1-2 days
- 3-5 days
- 6-7 days

8.2. Have you gone for long periods of time (e.g., 8 or more waking hours) without eating anything at all in order to influence your weight or shape?

- 0 days
- 1-2 days
- 3-5 days
- 6-7 days

8.3. Has thinking about <u>food, eating or calories</u> made it very difficult to concentrate on things you are interested in (such as working, following conversation or reading)?

- 0 days
- 1-2 days
- 3-5 days
- 6-7 days

8.4. Has thinking about your <u>weight or shape</u> made it very difficult to concentrate on things you are interested in (such as working, following a conversation or reading)?

- 0 days
- 1-2 days
- 3-5 days
- 6-7 days

8.5. Have you had a definite fear that you might gain weight?

- 0 days
- 1-2 days
- 3-5 days
- 6-7 days

8.6. Have you had a strong desire to lose weight?

- 0 days

- 1-2 days
- 3-5 days
- 6-7 days

8.7. Have you tried to control your weight or shape by making yourself sick (vomit) or taking laxatives?

- 0 days
- 1-2 days
- 3-5 days
- 6-7 days

8.8. Have you exercised in a driven or compulsive way as a means of controlling your weight, shape or body fat, or to burn off calories?

- 0 days
- 1-2 days
- 3-5 days
- 6-7 days

8.9. Have you had a sense of having lost control over your eating (at the time that you were eating)?

- 0 days
- 1-2 days
- 3-5 days
- 6-7 days

8.10. On how many of these days (i.e. days on which you had a sense of having lost control over your eating) did you eat what other people would regard as an unusually large amount of food in one go?

- 0 days

- 1-2 days
- 3-5 days
- 6-7 days

8.11. Has your weight or shape influenced how you think about (judge) yourself as a person?

- Not at all
- Slightly
- Moderately
- Markedly

8.12. How dissatisfied have you been with your weight or shape?

- Not at all
- Slightly
- Moderately
- Markedly

8.13. What is your weight at present? (Please give your best estimate in kilograms)

- Free Text Response

8.14. What is your height? (Please give your best estimate in centimetres).

- Free Text Response

9. Hospital Anxiety and Depression Scale (HADS)

Select the response that is closest to how you have been feeling in the past week. Don't take too long over you replies: your immediate is best.

9.1. I feel tense or wound up

- Most of the time

- A lot of the time
- Occasionally
- Not at all

9.2. I still enjoy the things I used to enjoy

- Definitely as much
- Not quite as much
- Only a little
- Hardly at all

9.3. I get a frightened feeling as if something awful is about to happen

- Definitely as much
- Not quite as much
- Only a little
- Hardly at all

9.4. I can laugh and see the funny side of things

- As much as I always could
- Not quite so much now
- Definitely not so much
- Not at all

9.5. Worrying thoughts go through my mind

- A great deal of the time
- A lot of the time
- Not too often
- Only occasionally

9.6. I feel cheerful

- Not at all

- Not often
- Sometimes
- Most of the time

9.7. I can sit at ease and feel relaxed

- Definitely
- Usually
- Not often
- Not at all

9.8. I feel as if I am slowed down

- Nearly all the time
- Very often
- Sometimes
- Not at all

9.9. I get a frightened feeling like ‘butterflies’ in the stomach

- Not at all
- Occasionally
- Quite often
- Very often

9.10. I have lost interest in my appearance

- Definitely
- I don’t take as much care as I should
- I may not take quite as much care
- I take as much care as ever

9.11. I feel restless as if I have to be on the move

- Very much indeed

- Quite a lot
- Not very much
- Not at all

9.12. I look forward with enjoyment to things

- As much as I ever did
- Rather less than I used to
- Definitely less than I used to
- Hardly at all

9.13. I get sudden feelings of panic

- Very often
- Quite often
- Not very often
- Not at all

9.14. I can enjoy a good book or a radio or TV programme

- Often
- Sometimes
- Not often
- Very seldom

10. Attitudes Toward the ANZAED Eating Disorder Credential

Whether or not you have you received eating disorder treatment from an ANZAED credentialed clinician, please take the time to answer the following questions.

10.1. After you have read each question, please drag the slider to the point that most accurately reflects the extent to which you agree with the following statements:

10.1.1. In terms of the success of therapy, it makes no difference whether I receive treatment from a credentialed or non-credentialed clinician.

Strongly Disagree Strongly Agree

10.1.2. I would prefer receiving treatment from a credentialed clinician over a non-credentialed clinician.

Strongly Disagree Strongly Agree

10.1.3. I place greater trust in the advice of a credentialed eating disorder clinician than a non-credentialed clinician.

Strongly Disagree Strongly Agree

10.1.4. I value the existence of a Credential that recognises the expertise and training of clinicians and requires a commitment to engagement in continual professional development and supervision specific to the treatment of eating disorders.

Strongly Disagree Strongly Agree

10.1.5. The Credential will make it easier for people with an eating disorder to readily access specialised care.

Strongly Disagree Strongly Agree

10.1.6. The Credential will improve the health outcomes of eating disorder patients.

Strongly Disagree Strongly Agree

10.2. The following questions ask about your experiences of the ConnectED website (https://connected.anzaed.org.au). This website is designed to assist with access to Credentialed Eating Disorder Clinicians.

10.2.1. Have you used this website before?

- Yes
- No

Questions 10.2.2.-10.2.8. shown only to participants who answered "Yes":

10.2.2. How did you first hear about the Connect·ED website?

- From a health care professional
- From a friend/peer
- On the internet
- Other (please specify):

After you have read each question please drag the slider to the point that most accurately reflects the extent to which you agree with the following statements:

10.2.3. The ConnectED website is easy to navigate and useful for locating eating disorder treatment service providers.

Strongly Disagree Strongly Agree

	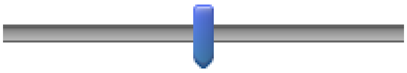

10.2.4. The information in clinician profiles was detailed and clear such that I felt confident to select a clinician who was the 'right fit' for me.

Strongly Disagree Strongly Agree

	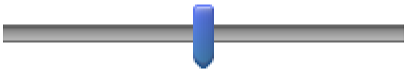

10.2.5. The search filters helped me to easily identify a clinician who was the 'right fit' for me.

Strongly Disagree Strongly Agree

	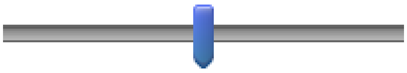

10.2.6. I felt that I could trust the information presented on the ConnectED website.

Strongly Disagree Strongly Agree

10.2.7. The ConnectED website used appropriate, respectful and inclusive language.

Strongly Disagree Strongly Agree

10.2.8. I would recommend the ConnectED website to other patients, carers and families who are seeking specialised treatment for an eating disorder.

Strongly Disagree Strongly Agree

11. Perceived barriers to seeking and accessing treatment questionnaire (Hamilton et al., 2022).

Please rate how each of the following barriers affected your access or use of outpatient or inpatient eating disorder treatment:

11.1. Cost (e.g. cost of service, travel expenses, time off work)

No impact at all *Moderately impactful* *Extremely impactful*

1 2 3 4 5

11.2. Stigma associated with eating disorder (e.g., did e.g., did not feel comfortable disclosing my condition to my GP, guilt or shame associated with my symptoms, fear of judgement from family/social group)

No impact at all		*Moderately impactful*		*Extremely impactful*
1	2	3	4	5

11.3. Inaccessible treatment (e.g., distance to treatment facility, long waitlist to get into treatment facility)

No impact at all		*Moderately impactful*		*Extremely impactful*
1	2	3	4	5

11.4. Social/work barriers (e.g., family obligations, work commitments)

No impact at all		*Moderately impactful*		*Extremely impactful*
1	2	3	4	5

11.5. GP/health eating disorder knowledge (e.g., GP/physician did not recognise the eating disorder)

No impact at all		*Moderately impactful*		*Extremely impactful*
1	2	3	4	5

11.6. Personal eating disorder knowledge (e.g., not knowing about treatments for eating disorders or where to find them)

No impact at all		*Moderately impactful*		*Extremely impactful*
1	2	3	4	5

11.7. Other, please specify: _________

No impact at all		*Moderately impactful*		*Extremely impactful*
1	2	3	4	5

FINISH

© **This survey is copyrighted to Janet Conti, Western Sydney University, and is not to be used or distributed without permission.**
