# Supplementary material for: The perceived impact of the ANZAED Eating Disorder Credential: perspectives of individuals with eating disorder lived experience
Source: J Eat Disord. 2026 Jul 31;13(Suppl 1):299. doi: 10.1186/s40337-026-01690-y (PMC13428425; doi:10.1186/s40337-026-01690-y)
Supplement: Supplementary file 2 — Supplementary Material 2: Author Reflexivity Statement. This file contains an author reflexivity statement detailing the clinical, academic, and methodological backgrounds of the research team and exploring how these positions influenced the data analysis and interpretation. [file 40337_2026_1690_MOESM2_ESM.docx]

**ADDITIONAL FILE 2:** Author reflexivity statement

Madalyn McCormack is a female, Anglo-Australian research officer, psychologist, and Credentialed Eating Disorder Clinician. Her clinical experience working with adults with eating disorders and other mental health concerns may have influenced her interpretation of participants narratives of their treatment experiences. Tracy Shand is an Anglo-Australian female clinical psychologist registrar working with ED clients. She experienced an ED as an adolescent which may have influenced data interpretation. Janet Conti is a female, Anglo-Australian clinician and researcher whose research is focused on innovating ED treatments through drawing on the wisdom of people living with an eating disorder and their support networks. Her interest in the broadening of treatment options for people experiencing EDs may have increased her responsiveness to participant quotes in this research that indicated the need for broadening the ED treatment options. Phillipa Hay is a cisgender female psychiatrist and Credentialed ED Clinician with a long-standing clinical and research commitment to improving the understanding of the experience of an eating disorder and its treatment.  She was formally trained first in psychodynamic psychotherapy and then in cognitive behaviour therapy. She has experience of caring for many people with eating disorders in general hospitals and outpatient private practice setting, has been a lead investigator on several clinical trials and lead author on Australian guidelines endorsing the need for new person centered and flexible approaches in care. Katarina Prnjak is a female provisional psychologist with 7 years of experience conducting research in the field of EDs. Her experience working in a multidisciplinary team increased responsiveness to quotes mentioning the value of teamwork. Rebecca Barns is a female, British clinical psychologist and researcher living and working in Australia. Her work focuses on trauma-informed and evidence-based approaches to eating disorders and related mental health difficulties, including integrating lived experience perspectives and body-based practices, such as yoga, into treatment and recovery frameworks. Her commitment to person-centred and embodied approaches to care may have shaped her attentiveness to participant narratives and interpretation of themes that highlighted the importance of safety, agency, relational attunement and connection within treatment, reflecting her belief in the value of holistic, flexible, and compassion-focused approaches in supporting recovery. Gabriella Heruc is a female dietitian and researcher with over 20 years working in the ED space. As the Credentialing Director for ANZAED, she was involved in the development and implementation of ANZAED and is passionate about ensuring its success. This may have increased her focus on responses about the perceived strengths of the Credential as well as areas for future improvement to support the Credential’s sustainability. Siân McLean is a cis-gender female, Anglo-Australian academic and researcher with 20 years of experience in eating disorders research. As a former President of ANZAED, she was involved in the development of the Credential and presently holds the volunteer position of co-chair of the Credentialing Governing Council. This may have focused her attention on participant responses that emphasised the differences in treatment experienced with credentialed and non-credentialed clinicians.
