# Supplementary material for: The perceived impact of the ANZAED Eating Disorder Credential: perspectives of individuals with eating disorder lived experience
Source: J Eat Disord. 2026 Jul 31;13(Suppl 1):299. doi: 10.1186/s40337-026-01690-y (PMC13428425; doi:10.1186/s40337-026-01690-y)
Supplement: Supplementary file 3 — Supplementary Material 3: Exemplar Data Extracts for Themes Identified from Participants Qualitative Survey Responses. This file contains exemplar data extracts for themes identified from participants qualitative survey responses. [file 40337_2026_1690_MOESM3_ESM.docx]

**ADDITIONAL FILE 3:** Exemplar Data Extracts for Themes Identified from Participants Qualitative Survey Responses

| **Themes** | **Exemplar Extracts** |
| --- | --- |
| Theme 1: Accessing consistent, expert care | *“Consistent access to support throughout the 8-week program (text support) + weekly sessions, real world experience and understanding from the counsellor providing hope and the 'other side' of recovery.”*  *“Intensity - realistically I required more intensive treatment, but this is few and far between in Tasmania.”*  *“Lack of medical support, my GP is not as experienced in managing eating disorders and I find it difficult to advocate for myself sometimes, especially as I am not currently underweight.”*  *“Lack of follow up after the therapy and referral to a psychiatrist who encouraged me to lose weight.”*  *“Just to get treatment without having to be underweight in my BMI, have treatment options available that are affordable or accessible. I stopped treatment due to the costs and my experience at the [removed hospital name to protect confidentiality] hospital when I had to go in for low potassium levels.”*  *“Doctor not understanding [removed service name to protect confidentiality] criteria for admission, basing severity of eating disorder on weight, comparing my weight to his as a measure of severity, ignoring physical observations and markers in criteria.”* |
| Theme 2: Working together in treatment | *“I would have liked my eating disorder team to listen to me and my needs and actually accommodate that, rather than stick to a one size fits all approach which definitely did not work for me.”*  *“The frequent referrals to 'recovery' and 'hope.' I often find the term 'recovery' puts a lot of pressure on me - like recovery is THE marvellous mecca to which I SHOULD be aspiring. I feel it sets me up to view recovery in terms of pass or fail, no in-between. Recovery, for me, suggests (because I am NOT recovered) that pinnacle I need to always be push, push, pushing towards - relentlessly - and anything less is a fault/shortcoming/inadequacy/failure on my part. It consequently can make my self-esteem just plummet - my confidence to - and actually kind of backfires, because it instils negativity within me.”*  *“Involuntary treatment was extremely difficult without a sense of autonomy, choice, power, control, etc.”*  *“Anecdotes were misogynistic and pushed that my appearance mattered. My health professional pushed that my main reason to restore my weight was to get back my period, which was only so I would be able to have babies and look desirable to men. He told me of what men find attractive in women, in a very objectifying fashion. This made me feel worthless and still does even now I'm weight restored.”*  *“A sense of partnership in my treatment between myself as a patient and my treatment team.”*  *“The psychologist refusing to speak to me, only my mum.”*  *“Having a team behind me and caring for me and instilling in me that recovery is possible has been such a significant blessing for me...”* |
| Theme 3: Feeling seen and supported in care | *“The nonjudgement and the caring nature of all involved has been paramount in me not giving up what little hope I have of recovery.”*  *“Being listened to, feeling understood, collaborative approach, being able to make changes slowly.”*  *“One size fits all treatment, no specific treatment for se-an sufferers.”*  *“The autonomy to make my own decisions, and recognition of my neurodivergence, which has been both empowering and informative.”*  *“Feeling respected and validated by my therapist.”*  *“Rigid rules, one size fits all approach to treatment, lack of 1:1 psychological input; psychiatrists influenced by pressure to discharge patients; [removed details of treatment to protect confidentiality] was highly unprofessional and openly criticised the role and place of Christian faith in supporting recovery.”*  *“I needed understanding. I felt so alone, and he made me feel like an outcast to a community I had expected to understand me or maybe empathise.”* |
